# Research into finding a stable prognosis parameter for the detection of students in need of guidance – Realization of equal opportunities through a diversity-oriented study guidance

**DOI:** 10.3205/zma001166

**Published:** 2018-05-15

**Authors:** Yassin Karay, Houda Hallal, Christoph Stosch

**Affiliations:** 1 University of Cologne, Medical Faculty, Dean's Office for Student Affairs, Cologne, Germany

**Keywords:** diversity, equal opportunities, study guidance, academic success, study progression

## Abstract

**Objective: **The internationalization of teaching and studying as well as increasing numbers of students with increasingly heterogeneous educational biographies and lifestyles require universities to develop awareness of this diversity and the need for adequate diversity management. For some diversity criteria at least it has been proven that they can influence the individual study success of students. The Dean’s Office of the Medical Faculty of the University of Cologne has empirically determined a stable prognosis parameter for study progression on the basis of selected criteria in order to enable early detection of students in need of guidance. This will then be used for targeted, diversity-oriented study guidance. On the one hand a correspondingly adapted guidance offer should take into account individual study progressions. On the other hand, measures to improve the equal opportunities of students with regard to their academic success can be discussed.

**Methodology: **With the help of study progression analyses, study progress of cohorts can be recorded longitudinally. The study progression analysis implemented in the control of faculty teaching serves as a central forecasting and steering tool for the forthcoming concept of diversity-oriented study guidance. The significance measurement of the various features is determined using binary logistic regression analyses.

**Results: **As part of the study progression analyses, the *study success rate after the first semester* has the strongest influence on the concordance with the minimum duration of study in the pre-clinical phase, followed by the characteristics *age at commencement of studies* and *place of university entrance qualification.* The school leaving grade only just misses the required significance level of p <0.05. As a predictor *gender* provides no explanatory contribution in the considered model.

**Conclusion:** In order to do justice to the heterogeneity among the students, university administrators and lecturers should understand the recognition of diversity as a cross-cutting task and keep an eye on diversity-related aspects and discrimination-critical topics for different target groups as well as individual guidance services in the context of individual study guidance. Within the scope of this study, we were able to empirically prove the stable prognosis parameter *study success rate after the first semester* allows reliable detection of students in need of guidance. The explanatory contribution is larger than any of the individual criteria examined in this study. The specific causes that led to a delay in studying will be analyzed in the context of downstream and diversity-oriented study guidance. A follow-up study will deal with the question of whether the success of students requiring study guidance can be significantly improved by subsequent study guidance.

## Zusammenfassung

**Zielsetzung: **Durch die Internationalisierung von Studium und Lehre sowie durch die ansteigenden Zahlen der Studierenden mit immer heterogeneren Bildungsbiographien und Lebensformen müssen Universitäten ein Bewusstsein für Diversität und die Notwendigkeit für ein adäquates Diverstiy-Management entwickeln. Es ist zumindest für einige Diversitätskriterien belegt, dass sie Einfluss auf den Studienerfolg von Studierenden nehmen können. Das Studiendekanat der Medizinischen Fakultät der Universität zu Köln hat, um beratungsbedürftige Studierende frühzeitig zu erkennen, einen stabilen Prognoseparameter zum Studienverlauf anhand ausgewählter Kriterien empirisch ermittelt. Dieser soll im Anschluss für eine gezielte, diversitätsorientierte Studienberatung eingesetzt werden. Ein entsprechend angepasstes Beratungsangebot soll zum einen individuellen Studienverläufen Rechnung tragen. Zum anderen können Maßnahmen zur Verbesserung der Chancengleichheit von Studierenden hinsichtlich ihres Studienerfolgs besprochen werden.

**Methodik: **Mit Hilfe von Studienverlaufsanalysen lassen sich Studienfortschritte von Kohorten longitudinal erfassen. Die im fakultären Lehrcontrolling implementierte Studienverlaufsanalyse dient dabei als zentrales Prognose- und Steuerungsinstrument für das neu einzuführende Konzept der diversitätsorientierten Studienberatung. Die Signifikanzmessung der verschiedenen Merkmale wird mit binär-logistischen Regressionsanalysen ermittelt.

**Ergebnisse:** Im Rahmen der Studienverlaufsanalysen liefert die *Studienerfolgsquote nach dem ersten Semester* den stärksten Einfluss auf die Einhaltung der Mindeststudienzeit im vorklinischen Abschnitt gefolgt von den Merkmalen *Alter bei Studienbeginn* und *Ort der Hochschulzugangsberechtigung*. Die Abiturnote verfehlt nur knapp das geforderte Signifikanzniveau von p<0,05. Der Prädiktor* Geschlecht* liefert im betrachteten Modell keinen Erklärungsbeitrag.

**Schlussfolgerung:** Um der Heterogenität bei den Studierenden gerecht zu werden, sollten Hochschulverwaltung und Hochschullehrende die Anerkennung von Vielfalt als Querschnittsaufgabe verstehen und im Kontext der individuellen Studienberatung diversitätsbezogene Aspekte und diskriminierungskritische Themen für unterschiedliche Zielgruppen sowie individuelle Beratungsangebote im Blick behalten. Im Rahmen dieser Studie konnte der stabile Prognoseparameter *Studienerfolgsquote nach dem ersten Semester* empirisch belegt werden und erlaubt eine zuverlässige Detektion beratungsbedürftiger Studierender. Der Erklärungsbeitrag ist größer als jedes einzelne in dieser Studie untersuchte Kriterium. Welche konkreten Ursachen zu einer Verzögerung des Studiums geführt haben, soll anschließend im Kontext einer nachgelagerten und diversitätsorientierten Studienberatung analysiert werden. Eine Folgestudie wird sich mit der Frage beschäftigen, ob durch eine nachgelagerte Studienberatung der Studienerfolg von beratungsbedürftigen Studierenden signifikant gesteigert werden kann.

## Introduction

Contrary to their official mission statements, higher education institutions often orient and organize themselves according to the “ideal of homogeneity of the so-called normal students” [1]; those normal students, “who are taking a first degree, are enrolled in a formal full-time study program, live away from home and are unmarried [2]”. However, this reference state is increasingly being questioned by higher education institutions, since the existing real heterogeneity of the students can be seen by the constant calls for an “explicit departure from the orientation towards so-called normal students” [1]. For if one casts a glance at the internationalization of study and teaching, and above all at the increasing number of students^1^ with increasingly varied educational biographies and forms of life, universities and their faculties must also develop an awareness of this diversity and adequate diversity management. For higher education, the question arises as to which diversity-oriented strategies and measures are needed in study guidance in order to enable students with different personal requirements to successfully complete their studies [3]. 

In order to fulfill the self-imposed mission statements, to “proactively engage in diversity, diversity of perspectives and equal opportunities”, an “inclusive” study environment must be able to map the individual requirements of the students. Only then can the universities create “framework conditions so they can be open to all people with appropriate access rights regardless of their circumstances and their social biographies”. Here, “the competent handling of diversity is understood as an enrichment and as a quality criterion” [4]. As the “diverse student learner” [5] replaces the “normal student”; this becomes deconstructed. This includes diversity-oriented study guidance. In order to explicitly make diversity useful for study guidance, a suitable university reference to a theoretical diversity model is needed. 

**Figure 1:**
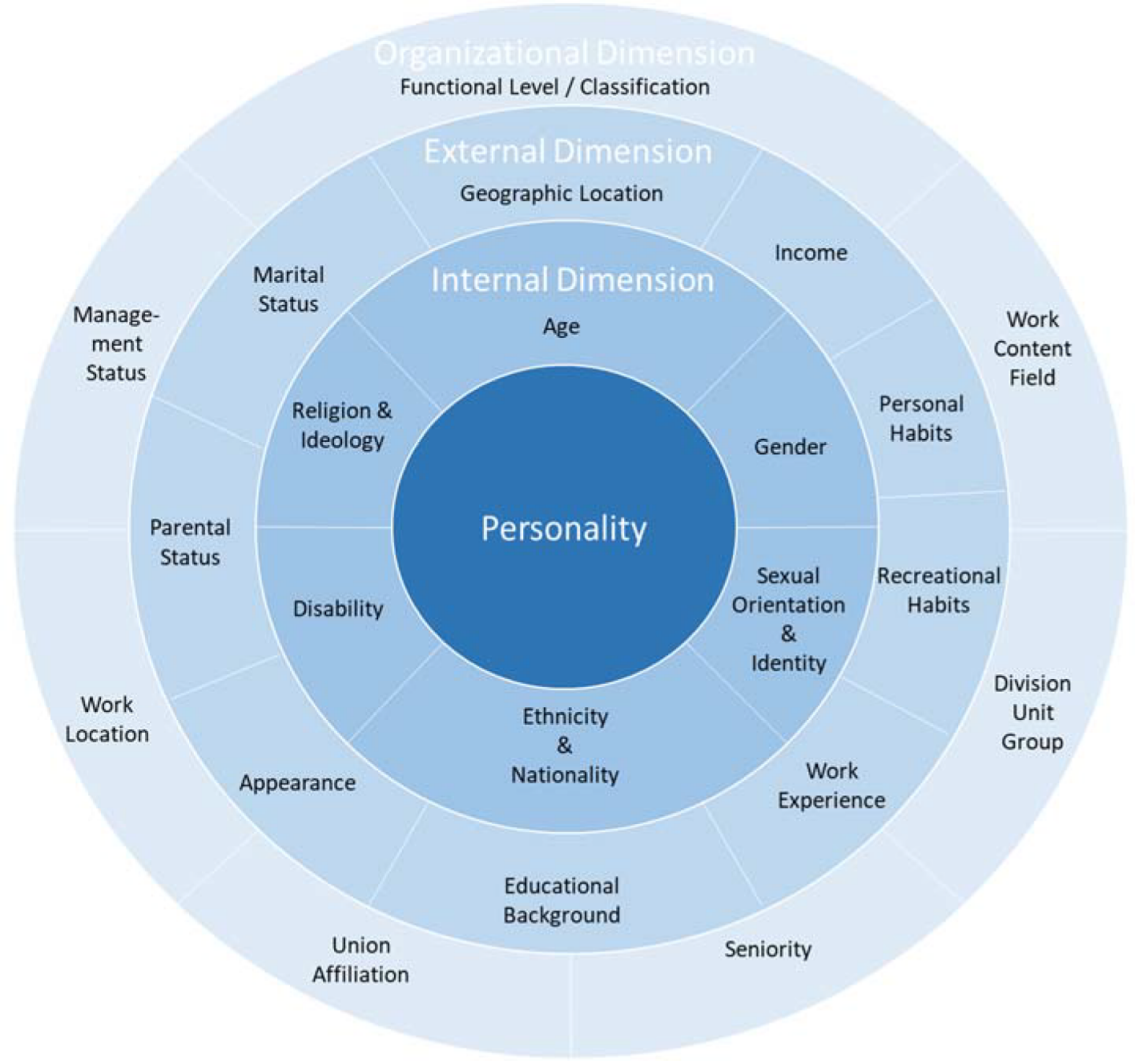
“Diversity Charter” based on Gardenswartz and Rowe

In our view, the four-dimensional diversity model (Four Layers of Diversity) by Gardenswartz and Rowe (2003) is a suitable reference [6]. At the center of Gardenswartz’s and Rowe’s four-dimensional diversity model is the personality followed by the almost unchangeable inner dimensions, such as age, gender, and nationality (see Figure 1 (Fig. 1)). 

The aspects of the third dimensional circle, such as marital status, income and education are referred to in the model as the external dimension. The organizational dimension with the features including place of work and duration of belonging complete the four-dimensional model. The external and organizational aspects of the respective institution can be flexibly and systemically adapted for diversity management. The four-dimensional diversity model of Gardenswartz and Rowe forms the core of the “Diversity Charter” and the diversity mission statement of the University of Cologne. The “Diversity Charter” is an organizational initiative that aims to “create a work environment that is free from prejudice” [7]. Of the 36 German universities with medical faculties, 21 have signed the “Diversity Charter” (own research, as of November 2017). Nationwide, 84 German universities have signed the “Diversity Charter”.

This theoretical positioning and nominal external and organizational diversity criteria are necessary because it is not uncommon for the multiplicity of existing diversity classifications and concepts to lead to a subjective perception, evaluation and prioritization of diversity categories. Above all, this approach can reduce the risk of focusing or “categorization using singular differences” [8]. Consequently, “the view is directed both to differences and similarities of structural disadvantage (...)” and hierarchies between the various diversity characteristics are avoided. “This requires a multi-dimensional understanding of diversity: Individual diversity characteristics are neither homogeneous nor do they occur in isolation. In addition, interactions may exist between them [9].” By incorporating institutional criteria into the model, the authors of the present article are able to identify, above all, those dimensions of diversity that are important for guiding higher education in medical faculties.

Against this background the following nominal, social-constructivist and statistically ascertainable diversity dimensions are defined:

- age and gender as classical dimensions of internal diversity
- institutionally relevant diversity criteria such as place of university entrance qualification as an indication of a domestic or foreign educational biography
- school-leaving grade as an indication of school education and
- the academic success rate after the first semester as an indication of the student’s ability to perform at university because, especially in the early study phase, the different educational biographies of the students play a special role [10].

The classical and well-known criterion “ethnicity/nationality” is replaced in this analytical context by the above-mentioned domestic or foreign educational biography. Since the so-called “migration background” has a selective effect, especially with regard to the second or subsequent generations [11], the disclosure and collection in this context would be misleading. A study which purely compares people with and without a migration background would result in an undifferentiated and unspecified perception. “The fact that not all migrants are identical, despite the simplification this expresses, cannot be stated often enough. Numerous operationalizations of the theoretical and empirical approaches of higher education research direct their attention to a different group of people, not only with regard to their migration status but also their position in academia” [12]. For the forecast model being developed, it is therefore more effective if “the variables (...) of the conditions of educational socialization” [13] are included. 

In the context of this study, the mentioned criteria are correlated with the successful completion of the pre-clinical phase because “equal opportunities” can be seen as a central requirement of the mission statement, especially in the study success parameter. In measuring study success, however, both a temporal and a success-related parameter should be used [14], in our case the successful completion of the basic medical examination in minimum study time. We have examined the central hypothesis whether a stable prognosis parameter for the detection of students in need of study guidance can be determined from the selected diversity criteria and provided for downstream, diversity-oriented study guidance, so that with focused guidance a mutually positive adjustment process with the goal of successful completion of studies, can be initiated. 

## Material and method

### Object of investigation

In Cologne, the study of human medicine, including exam preparation, consists of a total of twelve semesters plus three months. Students must study four semesters in the pre-clinical phase and six semesters in the clinical phase. Afterwards, the Practical Year (PY) and the final exams have to be completed successfully. This study focuses on the study time of the pre-clinical phase as an observation period so that study delays of individual students can be identified early on and in time.

In the Cologne model course, the assessment and exam results of students are administrated electronically in the university-wide campus management system. This creates the opportunity to follow the study progress of individual students and to evaluate them accordingly. Study progress of students can be longitudinally recorded with the help of electronically supported study course analyses so that individual and cohort-related minimum study time and withdrawal rates can be calculated [15]. 

#### Sample

For the study, six beginner cohorts were studied in greater detail, i.e. in total, 1,005 different educational biographies of students were analyzed with the help of the study course analyses. Three of the six beginner cohorts had begun their studies in the Summer Semester and three cohorts in the Winter Semester.

#### Variables 

Since the study delays of individual students are to be recognized early, the successful pass of the basic medical examination in minimum study time is used as a dependent variable (0=no, 1=yes). The basic medical examination in the Cologne model course corresponds to the first section of the medical exam according to the Medical Licensure Act (ÄApprO). The dependent variable therefore measures whether the pre-clinical phase was successfully completed after four semesters of minimum study time. This study quantifies the influence of students’ enrollment variables, such as gender, age at study entry, place of higher education entrance qualification (HZB), school-leaving grade and post-semester 1 student success rate on the binary variable regression analysis. In this way it is possible to determine dependencies between several independent variables.

The variables 1 to 5 in Table 1 (Tab. 1) provide information on those influencing factors identified as relevant in the introduction and which can be evaluated - based on the Four Layers of Diversity model of Gardenswartz and Rowe. Especially

1. gender,
2. age and
3. place of the HZB

can be evaluated as an indication of a domestic or foreign educational biography. The proportion of women in the whole sample is 60% (602 persons), which roughly corresponds to the proportion of women in the entire student body of human medicine in Cologne. The median age at commencement is 20.62 years and the mean is 22.5 years. Of the 1,005 students analyzed, 112 (11%) have gained their higher education entrance qualification abroad. As variables 4 and 5 provide information on the results of school education and also on university-level achievements, they are also examples and representative of the external dimension of education within the diversity model and can be used in the light of the present analysis. The mean of the school leaving grades is 1.6 and the median is 1.4. Overall, just under 60% of the students surveyed have a Leaving Certificate grade of 1.4 or better. After successful completion of semester 1, slightly less than half of the students surveyed had a 100% academic success rate after the first semester (47%, n=476). 529 students (53%) were already at least one assessment certificate behind after the first semester. 

Individual success and failure rates of students can be evaluated at any time due to the fixed semester plans in Cologne. The semester-based student success rate is calculated as the number of successfully completed assessments divided by the number of assessments proscribed by the semester plan. In Cologne, for example, the first semester of studies can therefore be divided into five roughly equivalent areas in terms of their duration [16] (see Table 2 (Tab. 2)).

## Result

In order to demonstrate the gradual evolution of the model coefficients, forward selection is used in the binary-variable regression analysis, so that the model coefficients are included in the model starting with the predictor with the highest explanatory power. Of the five independent features considered in total, three are shown to be significant predictors. The most important factor influencing compliance with the minimum study period is provided by the predictor* study success rate after semester 1*, as this is included in the model in the first step, followed by the variables *age at the start of study* and *place of university entrance qualification* (see Table 3 (Tab. 3)). In this model the characteristics of gender (p=0.944) and final leaving grade (p=0.076) had no significant influence on compliance with the minimum study time.

It can therefore be stated that the higher the student success rate after the first semester, the more likely it is that the minimum period of study will be observed (see also Table 4 (Tab. 4)). 

Of the students who achieved the required five assessment certificates in the first semester, a total of 78.8% completed the pre-clinical study phase within the minimum study time. For students with a pass rate of 80%, only one in two (51.5%) complies with the minimum study time, for students with three assessment certificates this figure is about one in five (19.8%) and in the group with less than three certificates only around 3.5%.

For the characteristic *age at commencement of studies*, younger undergraduates have better chances of success than older ones. The question if a student has attained university entrance qualification in Germany also has a significant impact on the compliance with the minimum study time.

The final model achieves a model quality of 0.491 as measured by the R^2^ value after Nagelkerke; 0.439 of the test quality are accounted for by characteristic* study success rate after Semester 1*, 0.04 by the characteristic *age at commencement* and 0.012 by location of the university entrance qualification as an indication of a domestic educational biography. The numbers confirm the high explanatory contribution of the study success rate after Semester 1 in the prognosis model under consideration, as almost 44% of the variance of the dependent variables (compliance with the minimum period of study in the pre-clinical phase) are explained by this characteristic alone. In total, almost 50% of the variance is described by the three significant characteristics study success rate after Semester 1, age at commencement and place of university entrance qualification. This can be interpreted as being good, a value above 0.5 is seen as a mark of very high model quality [17]. 

In the forecast model under consideration, the characteristic gender does not provide any noteworthy explanatory contribution to compliance with the minimum period of study in the pre-clinical phase. Accordingly, female and male students do not differ significantly in terms of compliance with the minimum period of study. The school leaving grade only just misses the required significance level of p <0.05. Statistically, the school leaving grade is overlaid by the study success rate after the first semester, i.e. although the school leaving grade reaches the required level of significance when these variables are removed but in the prognosis model with the significant predictors of age, place of university entrance qualification and school leaving grade, only a model quality R^2^ of only 0.187 after Nagelkerke. Compared to the above-mentioned final model with the parameter study success rate after Semester 1, 30.4% (0.304=0.491 minus 0.187) less of the variance of subordinate variables is explained. The predictor age at commencement of study is the parameter with the highest explanatory power, followed by the variables location of university entrance qualification and school leaving grade. 

## Discussion

The increasing number of students, with their heterogeneity in lifestyles, educational biographies and learning cultures, has led to a diversity in higher education in recent years, which has to be taken into account at the various levels of higher education and integrated into institutional processes. Practically speaking, this includes a systematic survey and evaluation of observable diversity characteristics of students and the answering the accompanying question of how this diversity can be supported institutionally by existing university courses. The goal must be to ensure diversity-sensitive equal opportunities for students with regard to their academic success. The focus primarily should be on the study entry phase [10]. Problems with complying with the minimum period of study can be detected at an early stage, after the first semester, by using a study progression analysis as presented here. 

In the context of this study, the highly predictive characteristic* student success rate after the first semester* shows just how essential the early period of studies is for subsequent study success. As the pre-clinical phase progresses, students may find it difficult to close the performance gap arising in the first semester, especially if their success rate is 60% or less. Having detected the performance issue in this group of students, intensive and individual study guidance seems advisable, because the causes for requiring guidance cannot be clarified in the context of a purely statistical investigation and must therefore be analyzed via a downstream, diversity-oriented study guidance.

The student guidance program of the Dean’s Office at the Medical Faculty of the University of Cologne therefore uses the highly predictive power of the study success rate after the first semester to estimate the individual guidance needs of students. The Faculty of Medicine expects this measure to enable the affected students to complete the pre-clinical phase with as little delay as possible, although participation in study guidance must remain voluntary (see Higher Education Act of North-Rhine Westphalia). In personal study guidance, it can be ensured that affected students are given the opportunity to present their individual study situation and to jointly explore the specific reasons causing the initial difficulties and hurdles. In addition to the characteristics already identified and analyzed above, this personal interaction with the students may also identify those characteristics which were mentioned in the introduction under the aspects of physical and mental abilities, marital status, socio-economic status, learning culture and which were not collected in this study progression analysis:

- If one considers, for example, the dimension “physical and psychological abilities”, it is important to note in diversity-sensitive study guidance that in the context of university life mental issues, for example in the field of interpersonal communication, manifest themselves in the form of exam anxiety, work disorders, decreased recall capacity, difficulty concentrating and reduced drive. The “mental abilities” must therefore be considered as factors which influence study progression in different ways. Against the background of the UN Convention on the Rights of Persons with Disabilities and Chronic Diseases of 2008, [18], this dimension must be taken into account insofar as mental abilities can have a relevant impact on the academic educational biography. Schaefer et al. have shown that among medical students, students with high exam anxiety suffered significantly more often from social and isolating fears than students with low exam anxiety [19]. In addition, students with high exam anxiety often suffered study delays.
- Numerous studies have already addressed the topic of possible influencing factors on the study behavior in medical studies within the framework of educational research and provide insights for further characteristics which inhibit or promote study success. For example, learning style [20], learning capacity [21], or personality traits such as conscientiousness [22] can have a positive or negative impact on study success. Other study programs for example have shown that identification with the study subject [23], occupational intensity [24], extrinsic and intrinsic study motivation [25], or the introduction of tuition fees [26] can significantly influence study success.
- Other hurdles which can only be identified in individual study guidance such as a low socio-economic status, lack of financial support or poor educational family background, can complicate normal and successful study progression.

The large number of possible influencing factors shows that a positive adaptation process can only be initiated on both sides through a downstream diversity-oriented study guidance service. This examination of students aims to systematically analyze the observable diversity characteristics, how they can be supported through existing university services or which measures have been or have to be taken in order to integrate them into the existing range of university services.

In the context of this study, for example, no significant impact of **gender** on compliance with the minimum period of study in the pre-clinical phase was found. This result corresponds to the disagreement amongst international studies, which rate the performance of female medical students different in comparison with their fellow male students. While the studies by Ferguson et al. [20] and Arulampalam et al. [27] find that female students graduate from medical school with better final grades and/or break off their studies less frequently than male medical students, a study at the Medical University of Vienna shows the opposite. There, male students perform better than their female counterparts, especially in the pre-clinical phase of their studies [21].

In this study the characteristic of the** final school leaving grade** only just misses the required significance level. This can be interpreted as having a variety of possible causes. On the one hand, the school leaving grade is superseded by the variable study success rate after the first semester, i.e. although the school leaving grade reaches the required significance level when these variables are removed, this results in an overall model quality (R^2^) of only 0.187 (see also the significance of average school leaving grades in medical studies by Kadmon et al. [28]) and, in all probability, on the other hand it is likely that the selection procedure used in Cologne up until the Summer Semester of 2016, and the associated entailed lack of relationship between school leaving grade subjects and university subjects, played a role in this result [29]. To give some additional background, in Germany, after setting aside certain quotas e.g. for foreign students, the medical study places are allocated as follows:

- 20% for students with the best school leaving grade
- 20% based on accumulated deferral period and
- 60% based on the selection criteria of the university

Universities may formulate their own selection criteria within the framework of admission quotas, however, the school leaving grade must remain a significant yardstick [30].

Because up until the Summer Semester 2016 in Cologne 80% of the students were admitted on the basis of their school leaving grade and 20% of students on the basis of their accumulated deferral period, the examined school leaving grades lay mostly between 1.0 and 1.4, so it was hardly possible to explain the variance in compliance with the standard period of study in the pre-clinical phase through the school leaving grades. The advanced courses of aspiring high school graduates were strategically selected primarily on the basis of being good grades - ignoring subject choices preparing for scientific subjects at university. However, it has repeatedly been shown that there is a compelling connection between the ability to fulfill pre-clinical requirements and the study of scientific subjects in school [31]. The Medical Faculty of the University of Cologne identified this knowledge gap in their students’ scientific knowledge through regular monitoring of the success rates in the subjects of biology, chemistry and physics and as a result regularly offers scientific pre-courses and physics and chemistry tutorials to accompany lectures, to facilitate bringing together the professional expectations of the teachers and the actual previous knowledge of the students. In addition, the Medical Faculty of the University of Cologne has opted for an additional criterion alongside the average school leaving grade in the university’s admissions process. Since the Winter Semester 2016/17, in addition to the school leaving grade (51%), the test result of the Test for Medical Degree Programs (TMS) is also taken into account during admission with a 49% weighting, as the TMS also examines basic medical and scientific understanding. Initial analyses of the cohorts of the Winter Semester 2016/17 and Summer Semester 2017 show that students admitted with TMS perform better in the science subjects of biology, chemistry and physics than students selected based on best school leaving grade.

In the context of this study it was possible to prove an **age effect** on the compliance with the minimum period of study. Specifically, younger undergraduates study faster than their older fellow students. Since about 20% of the nationwide medical study places are distributed through a deferral period quota, it can be surmised that a higher age at commencement of study indicates later admission due to poorer school leaving grades [32]. In addition, it can be assumed that a longer intermission between the end of schooling and the commencement of studies or longer schooling can also lead to longer study times [24], [26]. Specific causes can only be evaluated in the context of diversity-oriented study guidance. For students with children the Office of the Dean of Studies offers early registration for courses with group teaching, so that the students can elect course times that are compatible with childcare before the official start of registration.

The analyses into the influence of the **place of the university entrance qualification** of the Leaving Certificate have shown that students with a German Abitur, which points to socialization within the German education system, study much faster and tend to comply with the minimum period of study more often than their fellow students who, having non-German Leaving Certificates and thus differing educational socialization, perform worse overall. As stated at the outset, this differentiation primarily aims to explain that the specific path to university admission and the preceding explicit and implicit educational socialization in relation to students with a migrant background should be examined with much greater attention to the heterogeneity and peculiarities of the migrant group than plain questions regarding nationality or origin allow [12]. For example, to overcomes issues of mother tongue and the different experiences of educational socialization amongst overseas students, the Cologne Academic International Office in cooperation with the Medical Faculty of the University of Cologne has started the program “Studienstart International” for medical students from outside Europe who do not have adequate German language skills (but also lack cultural-institutional knowledge) to better prepare them for medical studies in Germany. This program is intended to help students to study quickly and successfully, to establish good contact with fellow students, the faculty and other international and German students, and to find their feet at the University of Cologne. For example, the Studienstart International program offers German language courses which build upon individual knowledge levels. 

One limitation of our study is certainly the restriction to Cologne and the peculiarities of the Cologne model study course. In addition, achieving study progress in degree courses with fixed semester plans, as is generally the case in medical degrees, is easier to realize than in the case of courses without specific time plans. In addition, it must be noted critically that only characteristics collected during enrollment were used for this study progression analyses. It should be noted in this context that an analysis of all criteria from the four-dimensional model of Gardenswartz and Rowe is theoretically possible but requires significant resources and should therefore be part of subsequent study guidance on the ground. Although only five diversity criteria were selected, nearly 50% of the variance of the dependent variables (compliance with the minimum period of study in the pre-clinical phase) is explained by the significant characteristics of the predictive model, which can therefore be interpreted as good. 

## Conclusion and outlook

With downstream diversity-oriented study guidance, a positive adaptation process can be initiated on both sides, with the aim of initiating a more conscious decision for or against studying medicine. Through such individual or CV-based study guidance, students gain better insights into the complex study structures of the traditional German higher education system. The university is concerned with the systematic evaluation of the observable diversity characteristics of the students and in what way they can be supported institutionally by existing university services in order for as many students as possible to successfully complete their degrees. In parting it should be said that this method of identifying students in need of study guidance and the effectiveness of downstream measures must be accompanied by an intervention study. As a consequence of this study, the university and the corresponding institutions must ask themselves the fundamental question of which diversity criteria require special attention in the medium and long term as regards study guidance, and whether this results in the need for a comprehensive change of perspective for the systematic university guidance.

## Notes

^1^ “In the Winter Semester (WS) 2015/16, 745,009 students were enrolled at the universities in North Rhine-Westphalia - in comparison to the Winter Semester 2005/06 this represents an increase of 58.4%. [...] Number of students at NRW universities continues to increase, 06.07.2016”. (Data base: IT.NRW, Hochschulen in Nordrhein-Westfalen: Statistik kompakt – Ausgabe 2014, Düsseldorf 2014, S. 6.)

## Competing interests

The authors declare that they have no competing interests. 

## Figures and Tables

**Table 1:**
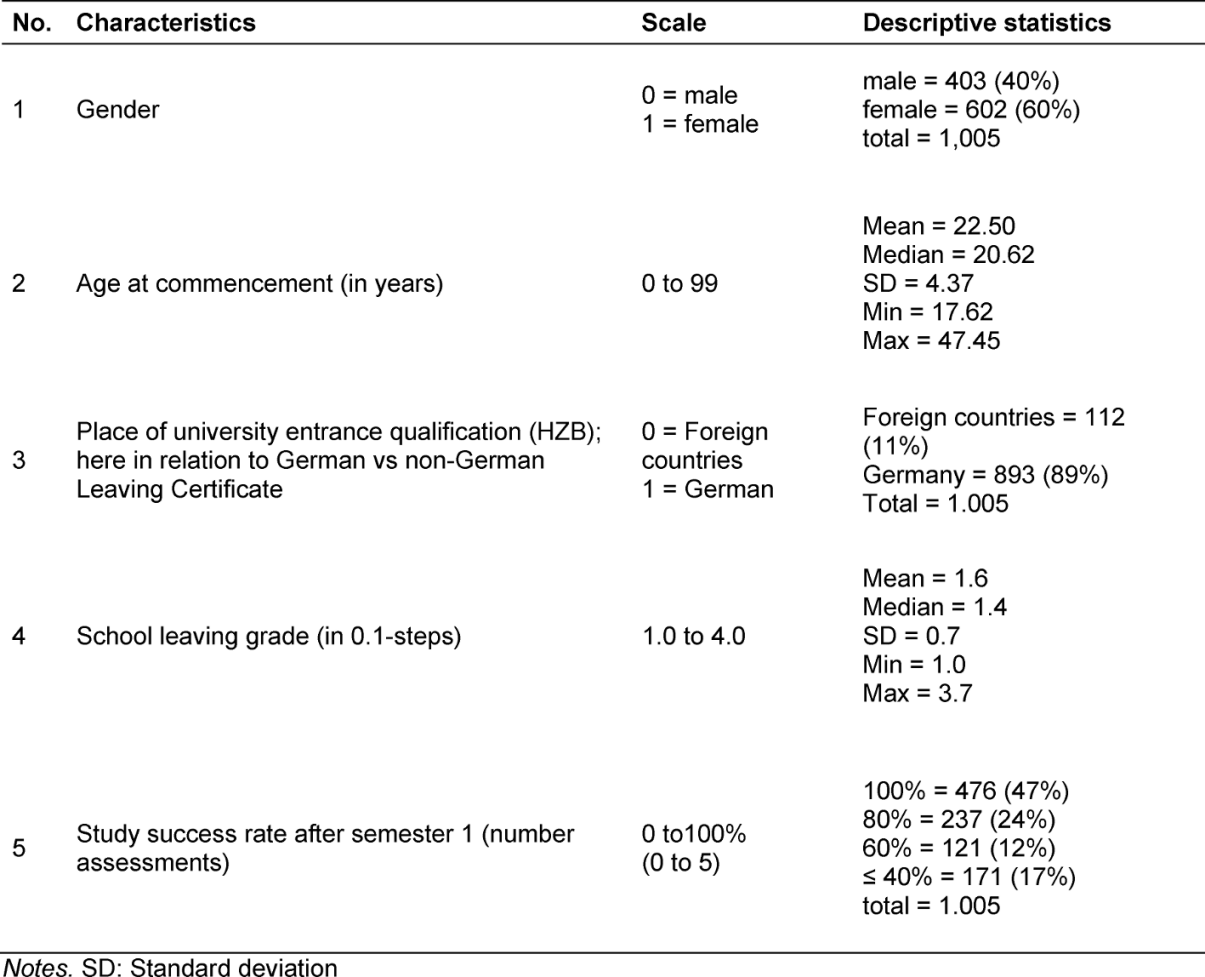
Features used to study a stable prognosis parameter

**Table 2:**
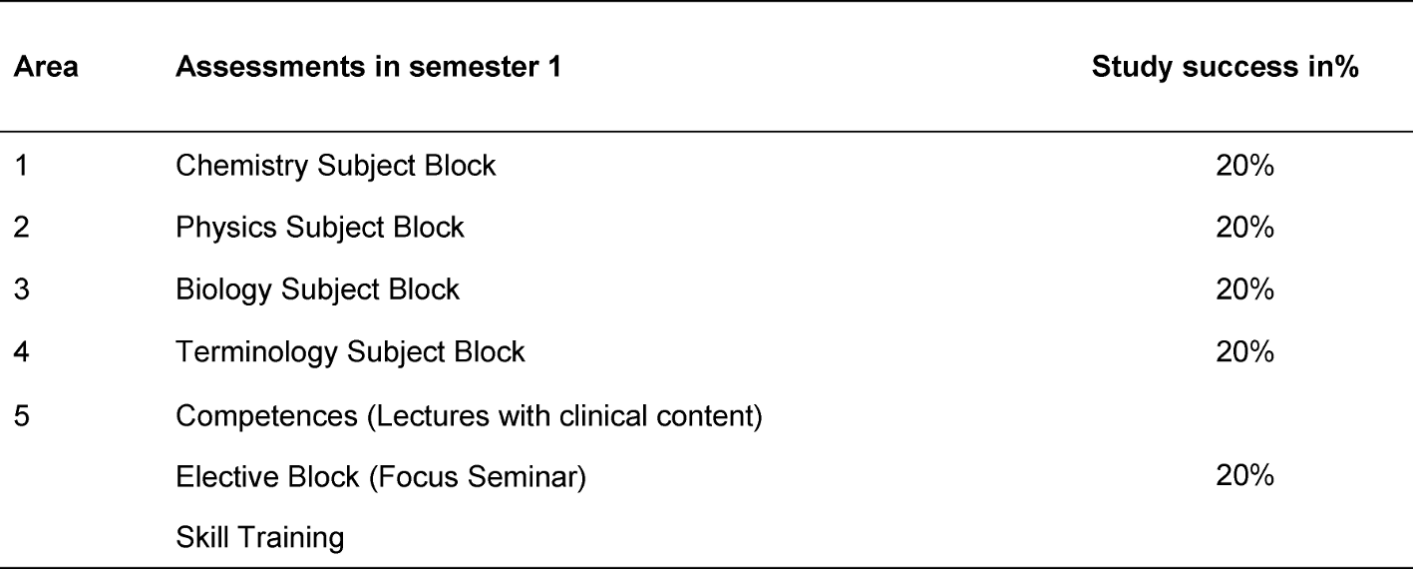
Assessments in the first semester (as of: Summer Semester 2017)

**Table 3:**
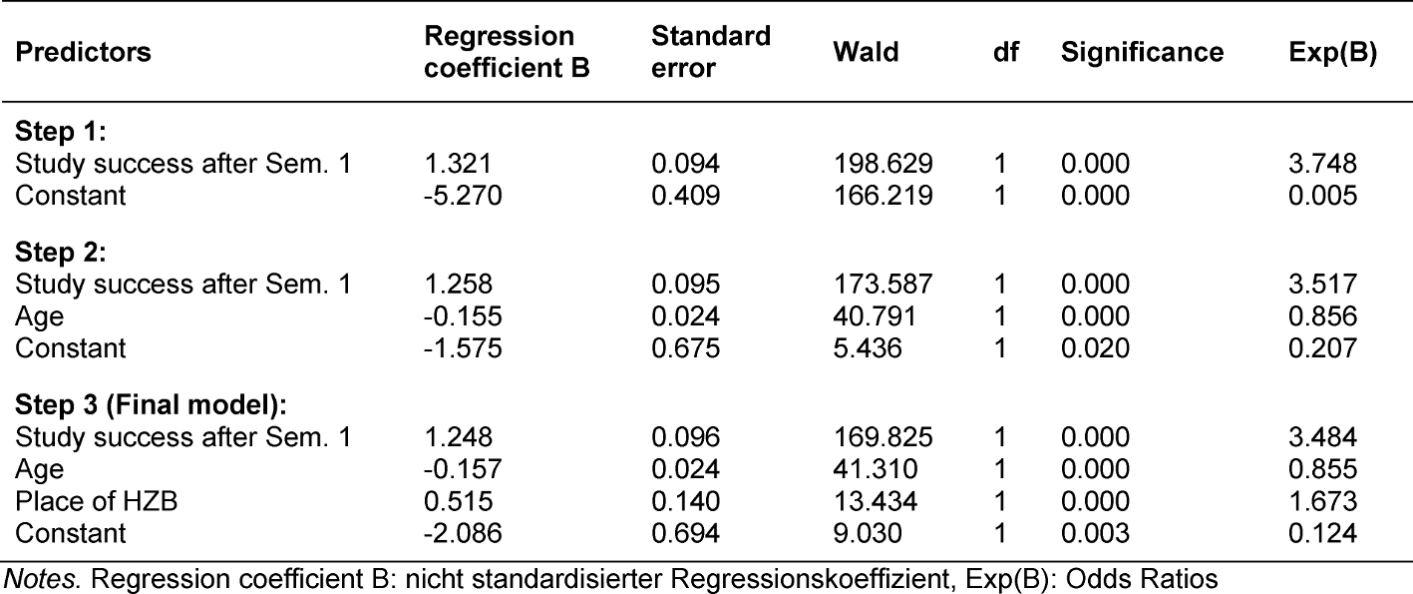
Significant Predictors

**Table 4:**
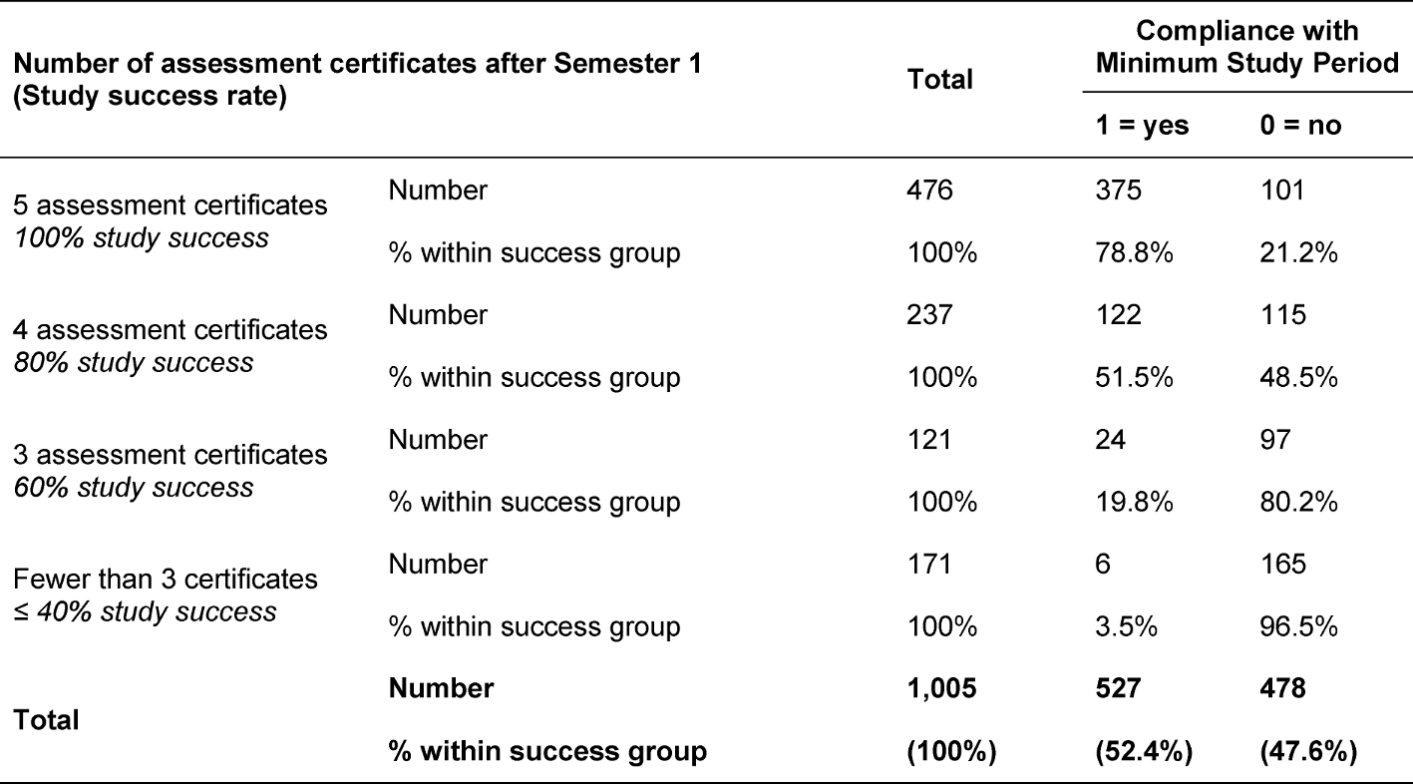
Study success rates after Semester 1
